# Effects of Intracoronary Pro-urokinase or Tirofiban on Coronary Flow During Primary Percutaneous Coronary Intervention for Acute Myocardial Infarction: A Multi-Center, Placebo-Controlled, Single-Blind, Randomized Clinical Trial

**DOI:** 10.3389/fcvm.2021.710994

**Published:** 2021-08-02

**Authors:** Dong Huang, Juying Qian, Zongjun Liu, Yawei Xu, Xianxian Zhao, Zengyong Qiao, Weiyi Fang, Li Jiang, Wei Hu, Chengxing Shen, Chun Liang, Qi Zhang, Junbo Ge

**Affiliations:** ^1^Department of Cardiology, Zhongshan Hospital, Fudan University, Shanghai, China; ^2^Department of Cardiology, Putuo District Central Hospital, Shanghai, China; ^3^Department of Cardiology, Tenth Hospital, Shanghai, China; ^4^Department of Cardiology, Changhai Hospital, Shanghai, China; ^5^Department of Cardiology, Fengxian District Central Hospital, Shanghai, China; ^6^Department of Cardiology, Shanghai Chest Hospital, Shanghai, China; ^7^Department of Cardiology, Tongren Hospital, Shanghai, China; ^8^Department of Cardiology, Minhang District Central Hospital, Shanghai, China; ^9^Department of Cardiology, Sixth Hospital, Shanghai, China; ^10^Department of Cardiology, Changzheng Hospital, Shanghai, China; ^11^Department of Cardiology, Shanghai East Hospital, Shanghai, China

**Keywords:** thrombolysis/thrombolytic agents, acute myocardial infarction, percutaneous coronary intervention, glycoprotein IIB/IIIA inhibitors tirofiban, myocardial reperfusion

## Abstract

**Background:** To determine whether intracoronary pro-urokinase or tirofiban improves myocardial reperfusion during primary percutaneous coronary intervention (PCI) for acute ST-segment elevation myocardial infarction (STEMI).

**Methods:** The study included patients with acute STEMI presenting within 12 h of symptoms at 11 hospitals in China between November 2015 and July 2017. Patients were randomized to receive selective intracoronary infusion of recombinant pro-urokinase (20 mg), tirofiban (10 μg/kg), or saline (20 mL) proximal to the infarct-related lesion over a 3-min period before stent implantation during primary PCI. The primary outcome was final corrected thrombolysis in myocardial infarction (TIMI) frame count (CTFC) after PCI.

**Results:** This study included 345 patients. Initial angiography identified a high-grade thrombus (TIMI 4–5) in 80% of patients. Final CTFC after PCI was significantly lower in the pro-urokinase (*P* < 0.001) and tirofiban (*P* < 0.001) groups than in the saline group and similar between the pro-urokinase and tirofiban groups (*P* > 0.05). The pro-urokinase (*P* = 0.008) and tirofiban groups (*P* = 0.022) had more complete ST-segment resolution at 2 h and lower peak creatine kinase-MB levels after PCI than the saline group (*P* = 0.006 and *P* = 0.023). The 30-day incidence of major adverse cardiac events was 4.5% in the pro-urokinase group, 3.4% in the tirofiban group, and 2.6% in the saline group. The incidence of in-hospital TIMI major bleeding events was low and comparable between groups.

**Conclusions:** Adjunctive intracoronary pro-urokinase or tirofiban given before stent implantation during primary PCI improves myocardial reperfusion without increasing the incidence of major bleeding events.

## Introduction

Ischemic heart disease (IHD) due to atherosclerosis is an important cause of morbidity and hospitalization worldwide (1) and responsible for around 9 million deaths annually (2). Rupture of an atherosclerotic plaque and subsequent thrombus formation leads to a reduction in blood flow through a coronary artery that causes acute coronary syndrome, which is a disease spectrum that includes ST-segment elevation myocardial infarction (STEMI). The prevalence of STEMI has increased nearly 4-fold in China during the last decade due to population aging and lifestyle changes, and the in-hospital mortality rate is around 10% (3, 4). Risk factors associated with IHD include age, gender, smoking, family history of IHD, hypertension, diabetes mellitus, obesity, increased level of low-density lipoprotein-cholesterol, increased level of triglycerides, and decreased level of high-density lipoprotein-cholesterol (5, 6). Early myocardial revascularization is key to the treatment of STEMI (7), and the revascularization procedures available include coronary artery bypass grafting and percutaneous coronary intervention (PCI) (8).

Primary PCI to reopen the occluded coronary artery is the evidence-based standard of care for patients with acute STEMI (9). However, myocardial reperfusion following primary PCI is often suboptimal due to distal thrombus embolization that impairs microvascular reperfusion and increases infarct size, especially in patients with a high thrombus burden (10–12). Glycoprotein IIb/IIIa inhibitors such as tirofiban prevent platelet aggregation and have been administered intravenously to reduce periprocedural ischemic events in patients undergoing PCI (13). Notably, studies during the last decade have suggested that intracoronary administration of a glycoprotein IIb/IIIa inhibitor during primary PCI can improve myocardial reperfusion and clinical outcomes in patients with STEMI (14–19).

Thrombolytic therapy with agents such as streptokinase and alteplase (also known as tissue plasminogen activator) can be used as an alternative to PCI or in combination with PCI for the management of STEMI (20). Several studies have reported that intracoronary administration of streptokinase or alteplase immediately after primary PCI can improve myocardial reperfusion and limit infarct size (21–23). Thus, there is growing interest in the potential use of adjunctive intracoronary fibrinolytic therapy during primary PCI (24). Like saruplase, recombinant human pro-urokinase (pro-UK) has structural similarities to alteplase and can be converted into active urokinase on the thrombus surface to produce thrombolytic effects (25, 26). However, to our knowledge, no previous investigations have evaluated the potential benefits of administering intracoronary pro-UK during primary PCI.

We hypothesized that intracoronary administration of pro-UK during primary PCI would enhance coronary flow in patients with STEMI. Therefore, the aim of this prospective, randomized clinical trial was to compare the effects of intracoronary pro-UK and intracoronary tirofiban on myocardial reperfusion in patients with STEMI undergoing primary PCI.

## Materials and Methods

### Study Design and Participants

The ERUPTION trial was a multi-center, prospective, randomized, placebo-controlled, single-blind trial. The current study enrolled acute STEMI patients presenting within 12 h of symptoms from 11 centers in China between November 2015 and July 2017. The inclusion criteria were: prolonged chest pain, persistent ST-segment elevation, and a clinical diagnosis of acute STEMI; presented within 12 h of symptom onset; and Thrombolysis in Myocardial Infarction (TIMI) coronary flow grade 0 (no flow), 1 (penetration without perfusion), or 2 (partial perfusion) in a major coronary artery. The exclusion criteria were: rescue PCI after thrombolytic therapy; age <18 years; need for emergency coronary artery bypass grafting; presence of cardiogenic shock; inability to provide informed consent; and contraindications for the use of tirofiban or thrombolysis including active internal bleeding, history of intracranial hemorrhage or ischemic stroke within 6 months, recent major surgery or trauma, severe uncontrolled hypertension, thrombocytopenia and severe liver or kidney failure. All patients provided written informed consent, and the study protocol was approved by the ethics committee of Zhongshan Hospital affiliated to Fudan University (No. B2012-134). The study was performed in accordance with the Declaration of Helsinki and Good Clinical Practice guidelines and is registered at ClinicalTrials.gov (NCT02131220).

### Randomization and Grouping

Upon admission, all patients were administered aspirin 300 mg and ticagrelor 180 mg or clopidogrel 600 mg. After the completion of coronary angiography, patients meeting the eligibility criteria were randomly assigned to one of three treatment groups (pro-UK, tirofiban or control) at a ratio of 1:1:1 using central randomization and opaque sealed envelopes. Randomization was stratified by clinical center, and block randomization for each center was performed with a block size of three. The randomization center prepared the opaque sealed envelopes containing the treatment group information according to the random number table generated by SAS9.3 software (SAS Institute, Cary, NC, USA). The investigator assigned the appropriate study drug (pro-UK, tirofiban or saline) to each participant according to the information contained in the envelope. Each center was required to file the randomization envelope so that it was available for later inspection.

### Interventions and Blinding

The patients were randomly assigned to receive one of three treatments: pro-UK (20 mg dissolved in 20 mL sterile water for injection; Tasly, Tianjin, China), tirofiban (10 μg/kg dissolved in 20 mL sterile water for injection; GrandPharma, Wuhan, China), or normal saline (20 mL). The drug was prepared by a nurse and infused over a 3-min period through a selective intracoronary catheter proximal to the culprit lesion in the infarct-related artery. The infusion was carried out after reperfusion had been achieved using manual thrombus aspiration or balloon dilation and before stent implantation. The operator was not blinded to the treatment used, but the patient and independent data reviewer were blinded to the grouping.

Additional balloon dilatation and stent implantation procedures were performed at the discretion of the operator. During PCI, unfractionated heparin (70–100 U/kg) was administered as an anticoagulant. Standard therapy after PCI included aspirin, clopidogrel or ticagrelor, a β-blocker, a statin, and an angiotensin-converting enzyme inhibitor or angiotensin II receptor blocker in accordance with contemporary practice guidelines (9).

Angiograms were recorded at 30 frames/s. TIMI flow grade, myocardial blush grade (MBG) and corrected TIMI frame count were measured by an independent angiogram reviewer at the Angiographic Core Laboratory of Zhongshan Hospital. The angiogram reviewer was blinded to the treatment grouping.

### Measurement of Clinical Parameters

The standard 18-lead electrocardiogram was recorded on admission, immediately after PCI and subsequently at 2, 6, 12, and 24 h after PCI. ST-segment resolution was measured independently at the Core Electrocardiographic Laboratory of Zhongshan Hospital. ST-segment resolution was measured as the percent resolution of the summed ST-segment elevation in the infarct leads from paired electrocardiograms (before PCI and 2 h after PCI) and classified as complete (≥70%), partial (30 to 70%), or none (≤ 30%) (27). The concentrations of cardiac markers, including creatine kinase (CK)-MB and cardiac troponin T, were measured on admission and subsequently at 6, 12, 24, and 48 h after PCI. Transthoracic echocardiography was performed at 1 and 30 days after PCI, and left ventricular ejection fraction (LVEF) was measured by an independent observer blinded to the treatment allocation.

### Outcomes and Follow Up

The primary outcome was the final corrected TIMI frame count (CTFC) after PCI. Secondary outcomes of myocardial reperfusion included post-procedural TIMI flow grade, MBG, incidence of complete ST-segment resolution at 2 h after PCI, peak CK-MB level at 1 and 30 days after PCI, LVEF assessed by echocardiography at 1 and 30 days after PCI, and incidence of major adverse cardiac events (MACEs, defined as the composite of cardiac mortality, non-fatal myocardial reinfarction and target vessel revascularization) at 30 days after PCI.

The safety outcome was the incidence of in-hospital major bleeding events defined according to the TIMI bleeding classification (28), which were evaluated during the 30-day follow-up by a committee of three physicians who were blinded to the treatment allocation.

### Definitions

Reperfusion time was defined as the time from onset of chest pain to wire crossing. Target vessel revascularization was defined as ischemia-driven revascularization of the infarct-related artery with PCI or coronary artery bypass grafting. TIMI flow grade and MBG were defined as previously described (29, 30). The TIMI thrombus grading scale was used to quantify the thrombus burden after wire crossing: grade 0, no thrombus; grade 1, possible thrombus present; grade 2, small-sized thrombus ≤ ½ vessel diameter; grade 3, moderate-sized thrombus >½ but <2 vessel diameters; grade 4, large-sized thrombus ≥2 vessel diameters; grade 5, total occlusion (31). The scale was also simplified to a binary system: low-grade thrombus (TIMI 0–3) or high-grade thrombus (TIMI 4–5). CTFC, including the definition of the first frame and last frame, was determined according to the method described by Gibson et al. (32, 33). The frame counts of the left anterior descending artery were divided by 1.7 to correct for its longer length. Non-fatal myocardial reinfarction was defined as the recurrence of symptoms of ischemia with new ST-segment elevation and/or increases in the levels of cardiac markers (30).

### Sample Size

The study was designed to recruit a total of 330 patients based on the following assumptions: a power of 90% to detect a 20% reduction in CTFC in the pro-UK and tirofiban groups vs. the control group; a two-sided alpha level of 0.05; loss to follow-up of 10%; an allocation ratio of 1:1:1; and analysis requiring three pairwise comparisons. This calculation was based on the improvement in CTFC demonstrated for streptokinase vs. control in a previous study (22), which included participants who would have fulfilled the enrollment criteria for the present ERUPTION trial.

### Statistical Analysis

An intention-to-treat (ITT) analysis was performed using SPSS 15.0 (SPSS Inc., Chicago, IL, USA). Kolmogorov-Smirnov test was used to assess the normal distribution. Normally distributed continuous data are presented as mean ± standard deviation and non-normally distributed continuous data are described as median (interquartile range). One-way analysis of variance or Kruskal-Wallis Test was used for comparison between the three groups. Pairwise comparisons were made between groups by two-tailed Student's *t*-test (continuous data with normally distributed) or Mann-Whitney *U*-test (continuous data with non-normally distributed) (*P*-value is adjusted to <0.017). Categorical variables are presented Categorical variables are presented as *n* (%) and were compared between groups using the chi-squared test or Fisher's exact test. A two-sided *P* < 0.05 was taken to indicate statistical significance.

## Results

### Baseline Clinical Characteristics of the Study Participants

**Figure 1:**
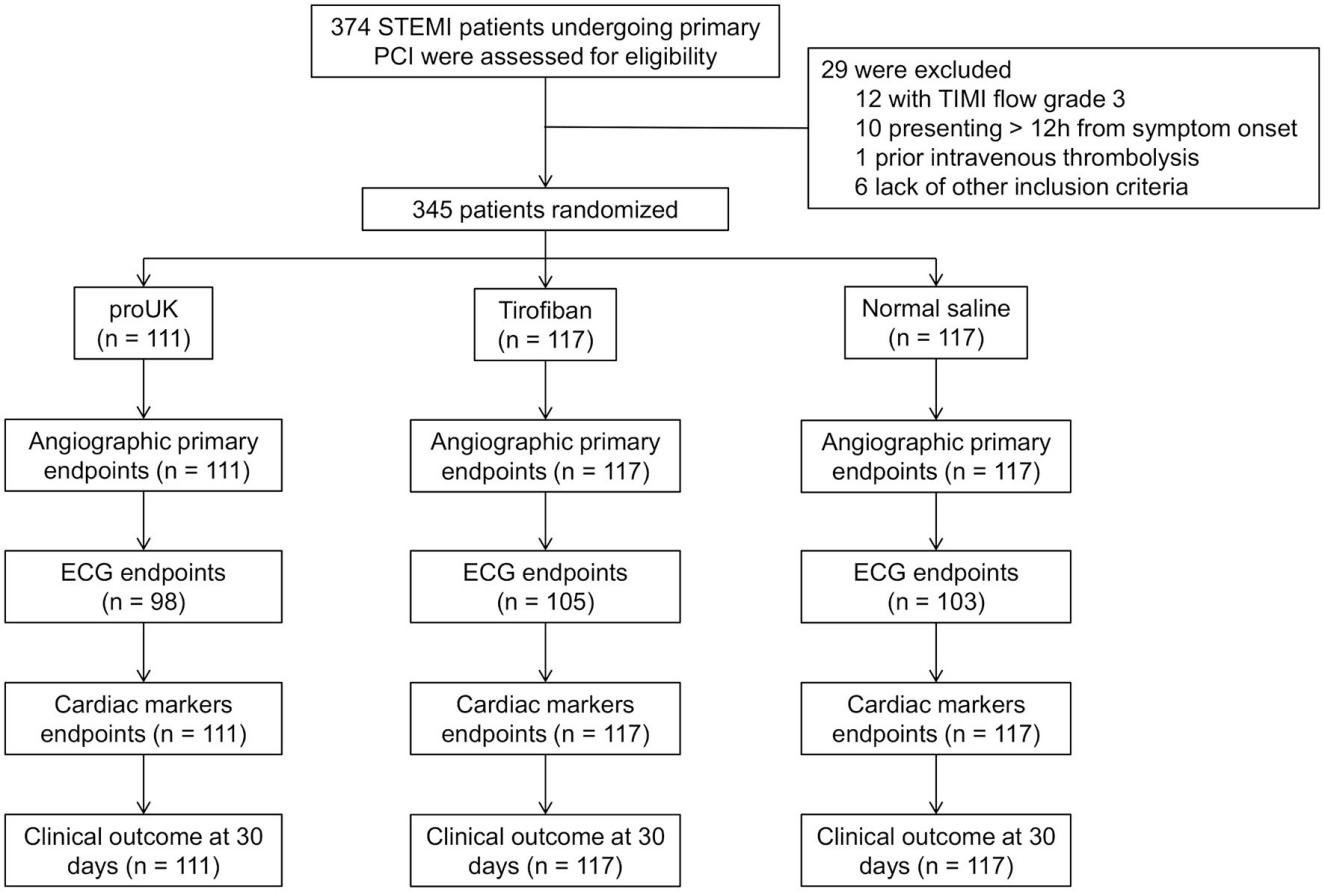
Flowchart showing patient enrollment. PCI, percutaneous coronary intervention; STEMI, ST-segment elevation myocardial infarction; TIMI, thrombolysis in myocardial infarction grade; ECG, electrocardiogram.

A total of 374 patients with STEMI scheduled for primary PCI were screened for inclusion, and 29 patients were subsequently excluded from the study (TIMI flow grade 3 in the infarct-related artery, *n* = 12; presented >12 h after symptom onset, *n* = 10; prior intravenous thrombolysis, *n* = 1; and failure to meet other inclusion criteria, *n* = 6). Therefore, 345 patients were randomly assigned to receive intracoronary infusion of pro-UK (*n* = 111), tirofiban (*n* = 117), or normal saline (*n* = 117) during PCI (Figure 1). There were no significant differences between the three groups in age, gender, body mass index, smoking history, medical history (hypertension, diabetes mellitus, dyslipidemia, previous myocardial infarction, or previous stroke), heart rate, systolic or diastolic blood pressure, infarct location, hemoglobin level, platelet count, or serum creatinine concentration (Table 1).

**Table 1:** Baseline clinical characteristics of the study participants.

**Characteristics**	**Pro-UK** **(*n* = 111)**	**Tirofiban** **(*n* = 117)**	**Control** **(*n* = 117)**	***P***
Age (years)	59.4 ± 10.1	60.6 ± 9.7	58.5 ± 9.9	0.286
Male gender	100 (90.1%)	101 (86.3%)	105 (89.7%)	0.607
Hypertension	52 (46.9%)	62 (52.1%)	58 (49.6%)	0.728
Diabetes mellitus	21 (18.9%)	27 (23.1%)	21 (18.0%)	0.583
Dyslipidemia	71 (64.0%)	74 (63.3%)	89 (76.1%)	0.064
Body mass index (kg/m^2^)	24.5 ± 2.5	25.3 ± 4.2	24.5 ± 2.5	0.236
Smoking history	67 (60.4%)	65 (55.6%)	75 (64.1%)	0.410
Previous MI	5 (4.5%)	1 (0.9%)	5 (4.3%)	0.210
Previous stroke	8 (7.2%)	8 (6.8%)	3 (2.6%)	0.228
Reperfusion time (hours)	3.9 ± 2.9	4.0 ± 2.6	4.3 ± 3.0	0.550
Systolic BP (mmHg)	121.1 ± 24.1	127.7 ± 24.7	121.2 ± 23.4	0.060
Diastolic BP (mmHg)	73.4 ± 15.1	77.7 ± 15.0	74.3 ± 13.9	0.075
Heart rate (beats/minute)	73.4 ± 19.6	74.4 ± 17.5	73.9 ± 17.4	0.918
Infarct location				0.845
Anterior	55 (49.5%)	58 (49.6%)	52 (44.4%)	
Inferior	46 (41.4%)	45 (38.5%)	51 (43.6%)	
Other	10 (9.0%)	14 (12.0%)	14 (12.0%)	
Hemoglobin level (g/dL)	146.6 ± 17.2	147.2 ± 14.4	148.7 ± 15.8	0.645
Platelet count (×10^9^/L)	213.0 ± 54.0	210.8 ± 67.4	223.5 ± 53.9	0.305
Creatinine (μmol/L)	78.8 ± 20.0	78.7 ± 21.2	74.3 ± 20.5	0.260

*Data are presented as mean ± standard deviation or n (%). BP, blood pressure; MI, myocardial infarction*.

### Procedural Characteristics

The procedural characteristics are summarized in Table 2. The mean reperfusion time was around 4 h and did not differ significantly between groups. Most of the procedures (~94%) were performed through radial artery access. Initial angiography demonstrated total occlusion of the infarct-related artery (TIMI flow grade 0) and the presence of high-grade thrombus (TIMI 4–5) in about 80% of the patients. Approximately half the patients underwent manual thrombus aspiration after wire crossing. Around 60% of the patients received a loading dose of ticagrelor, and the median dose of unfractionated heparin was 7,000 U. There were no significant differences between the three groups in any of the procedural characteristics.

**Table 2:** Procedural characteristics.

**Characteristics**	**Pro-UK** **(*n* = 111)**	**Tirofiban** **(*n* = 117)**	**Control** **(*n* = 117)**	***P***
Radial artery access	104 (93.7%)	111 (94.9%)	109 (93.2%)	0.855
**Infarct-related artery**				0.096
Left anterior descending artery	60 (54.1%)	60 (51.3%)	54 (46.2%)	
Left circumflex artery	7 (6.3%)	17 (14.5%)	20 (17.1%)	
Right coronary artery	44 (39.6%)	40 (34.2%)	43 (36.8%)	
**TIMI flow grade at initial angiography**				0.126
0	96 (86.5%)	92 (78.6%)	101 (86.3%)	
1	7 (6.3%)	12 (10.3%)	10 (8.5%)	
2	8 (7.2%)	13 (11.1%)	6 (5.1%)	
**TIMI thrombus grade after wire crossing**				0.335
0–3	9 (8.1%)	15 (12.8%)	7 (6.0%)	
4	6 (5.4%)	10 (8.5%)	9 (7.7%)	
5	96 (86.5%)	92 (78.6%)	101 (86.3%)	
Thrombus aspiration	58 (52.3%)	59 (50.4%)	58 (49.6%)	0.919
Balloon predilation	90 (81.1%)	96 (82.1%)	103 (88.0%)	0.300
Stent implantation	108 (97.3%)	111 (94.9%)	113 (96.6%)	0.611
Stent diameter (mm)	3.1 ± 0.4	3.1 ± 0.5	3.2 ± 0.5	0.520
Stent length (mm)	26.6 ± 7.5	25.4 ± 7.1	26.0 ± 7.6	0.376
Loading dose of aspirin	108 (97.3%)	114 (97.4%)	112 (95.7%)	0.712
**Loading dose of P**_**2**_ **Y**_**12**_ **antagonist**				0.619
None	3 (2.7%)	2 (1.7%)	1 (0.9%)	
Clopidogrel	43 (38.7%)	47 (40.2%)	39 (33.3%)	
Ticagrelor	65 (58.6%)	68 (58.1%)	77 (65.8%)	
Unfractionated heparin (U)	7,000 (5,000–9,000)	7,000 (5,000–8,500)	7,000 (5,500–9,000)	0.651

*Data are presented as mean ± standard deviation, median (interquartile range) or n (%). CTFC, corrected thrombolysis in myocardial infarction frame count; TIMI, thrombolysis in myocardial infarction grade*.

### Efficacy Analysis

The primary and secondary outcomes are compared between groups in Table 3. The final CTFC after PCI (primary outcome) was significantly lower (i.e., better) in the pro-UK group (*P* < 0.001) and tirofiban group (*P* < 0.001) than that in the control group. However, the final CTFC did not differ significantly between the pro-UK and tirofiban groups.

**Table 3:** Primary and secondary outcomes for the efficacy analyses.

**Characteristics**	**Pro-UK** **(*n* = 111)**	**Tirofiban** **(*n* = 117)**	**Control** **(*n* = 117)**	***P***
**Primary outcome**				
CTFC after PCI (frames)	27.1 ± 14.2*	28.8 ± 17.1*	34.6 ± 18.3	<0.001
**Secondary outcomes**				
TIMI flow grade after PCI				0.018
0	1 (0.9%)	0 (0.0%)	1 (0.9%)	
1	4 (3.6%)	3 (2.6%)	5 (4.3%)	
2	5 (4.5%)	8 (6.8%)	21 (17.9%)	
3	101 (91.0%)*	106 (90.6%)*	90 (76.9%)	
MBG after PCI				0.054
0–1	37 (33.3%)	38 (32.5%)	57 (48.7%)	
2	36 (32.4%)	38 (32.5%)	34 (29.1%)	
3	38 (34.2%)	41 (35.0%)	26 (22.2%)	
ST-segment resolution at 2 h after PCI (*n* = 306)				0.019
Complete	64 (65.3%)*	66 (62.9%)*	45 (43.7%)	
Partial	20 (19.6%)	24 (22.9%)	36 (35.0%)	
None	14 (14.2%)	15 (14.3%)	22 (21.4%)	
Peak CK-MB level (U/L)	307.8 ± 183.4*	376.6 ± 268.0*	442.5 ± 426.1	0.010
**Echocardiographic LVEF**				
Day 1 (*n* = 323)	52.5 ± 9.0	52.9 ± 9.2	53.3 ± 9.0	0.804
Day 30 (*n* = 285)	58.2 ± 7.8	57.1 ± 8.6	55.6 ± 8.6	0.218
**Clinical outcome at 30 days**				
Mortality	3 (2.7%)	3 (2.6%)	2 (1.7%)	0.863
Cardiac mortality	2 (1.8%)	2 (1.7%)	1 (0.9%)	0.802
Non-fatal myocardial reinfarction	2 (1.8%)	2 (1.7%)	1 (0.9%)	0.802
Target vessel revascularization	1 (0.9%)	0 (0.0%)	1 (0.9%)	0.596
MACEs	5 (4.5%)	4 (3.4%)	3 (2.6%)	0.726

Data are presented as mean ± standard deviation or n (%). CK-MB, creatinine kinase-MB; CTFC, corrected thrombolysis in myocardial infarction frame count; MACEs, major adverse cardiac events; MBG, myocardial blush grade; PCI, percutaneous coronary intervention; TIMI, Thrombolysis in myocardial infarction grade. * P < 0.05 vs. control group.

The distribution of MBG was borderline significant in favor of patients randomized to the pro-UK and tirofiban groups (*P* = 0.054). Moreover, the incidence of MBG 2/3 was significantly higher in the pro-UK group (66.6%, *P* = 0.022) and tirofiban group (67.5%, *P* = 0.016) than in the control group (51.3%). The percentage of patients achieving final TIMI grade 3 flow after PCI was also significantly higher in the pro-UK group (91.0%, *P* = 0.015) and tirofiban group (90.6%, *P* = 0.035) than that in the control group (76.9%). The proportions of patients with MBG 2/3 and final TIMI grade 3 were not significantly different between the pro-UK and tirofiban groups.

A full set of electrocardiographic data were available for 98 patients in the pro-UK group (88.3%), 105 patients in the tirofiban group (89.7%) and 103 patients in the control group (88.0%). The proportion of patients with complete ST-segment resolution at 2 h after PCI was significantly higher in the pro-UK group (65.3%, *P* = 0.008) and tirofiban group (62.9%, *P* = 0.022) than in the control group (43.7%) but was comparable between the pro-UK and tirofiban groups.

Peak CK-MB level of patients was significantly lower in the pro-UK group (307.8 U/L, *P* = 0.006) and tirofiban group (376.6 U/L, *P* = 0.023) than that in the control group (442.5 U/L). There was no significant difference in the peak CK-MB level between the pro-UK and tirofiban groups.

There were no significant differences between groups in LVEF at day 1 and day 30 after PCI. A total of 8 patients (2.3%) died during the 30-day follow-up period. All-cause mortality did not differ significantly between the pro-UK group (2.7%), tirofiban group (2.6%) and control group (1.7%). The incidence of MACEs was low and not significantly different between the pro-UK group (4.5%), tirofiban group (3.4%), and control group (2.6%).

### Safety Analysis

Safety data are presented in Table 4. The incidences of in-hospital TIMI major and minor bleeding were low and comparable between the pro-UK group (1.8 and 1.8%, respectively), tirofiban group (0.9 and 1.7%, respectively), and control group (0 and 0.9%, respectively). The incidence of TIMI minimal bleeding was numerically higher in the pro-UK group (13.5%) than in the tirofiban group (6.8%) or control group (6.0%), although the differences were not statistically significant (*P* = 0.078). One patient in the pro-UK group died from intracranial hemorrhage.

**Table 4:** Safety outcomes in-hospital bleeding outcomes.

**Bleeding outcome**	**Pro-UK** **(*n* = 111)**	**Tirofiban** **(*n* = 117)**	**Control** **(*n* = 117)**	***P***
TIMI major	2 (1.8%)	1 (0.9%)	0 (0.0%)	0.343
TIMI minor	2 (1.8%)	2 (1.7%)	1 (0.9%)	0.802
TIMI minimal	15 (13.5%)	8 (6.8%)	7 (6.0%)	0.078
TIMI major and minor	4 (3.6%)	3 (2.6%)	1 (0.9%)	0.552
Intracranial hemorrhage	1 (0.9%)	0 (0.0%)	0 (0.0%)	0.349

*Data are presented as n (%). TIMI, Thrombolysis in myocardial infarction grade*.

## Discussion

An important finding of the present study was that patients with STEMI receiving adjunctive intracoronary pro-UK or tirofiban during PCI had a better post-procedural CTFC than patients administered normal saline as a control. Furthermore, the pro-UK and tirofiban groups had more complete ST-segment resolution at 2 h and lower peak CK-MB level after PCI than the control group. The incidence of in-hospital TIMI major bleeding events was low and comparable between groups. Our results indicate that intracoronary administration of pro-UK or tirofiban during primary PCI for acute STEMI can improve myocardial reperfusion without increasing the rate of in-hospital major bleeding. To our knowledge, the ERUPTION study is the largest clinical trial to date to compare the effects of intracoronary fibrinolytic therapy with intracoronary administration of a glycoprotein IIb/IIIa inhibitor in patients with STEMI undergoing primary PCI, most of whom had a high thrombus burden and were treated using manual thrombus aspiration via radial artery access.

Distal embolization of thrombus from the culprit coronary artery during primary PCI can contribute to microvascular obstruction, and a high thrombus burden is associated with distal thrombus embolization and the no-reflow phenomenon during primary PCI. Glycoprotein IIb/IIIa inhibitors can prevent platelet aggregation and reduce thrombotic events in patients with acute coronary syndrome (34). Randomized clinical trials have suggested that intracoronary administration of tirofiban to achieve a high local concentration of the drug can reverse no-flow phenomena and attenuate microvascular obstruction in patients with STEMI undergoing primary PCI (19, 35–37). Our finding that myocardial reperfusion is improved by intracoronary infusion of tirofiban during primary PCI for acute STEMI is consistent with the above studies and with numerous other investigations evaluating the effects of intracoronary administration of glycoprotein IIb/IIIa inhibitors during PCI (14–19).

Theoretically, fibrinolytic therapy should be a more effective treatment for the fibrin-rich thrombi that undergo distal embolization and cause microvascular obstruction. The present study was designed to have sufficient power to detect whether intracoronary administration of pro-UK or tirofiban would improve final coronary flow when compared with a control group. We observed better outcomes for CTFC and MBG in patients randomized to pro-UK or tirofiban than in patients administered normal saline. The pro-UK and tirofiban groups also exhibited clinically relevant improvements in ST-segment resolution (an indicator of myocardial reperfusion) and peak CK-MB level (an indicator of infarct size) when compared with the control group, and these findings are consistent with those reported by Sezer et al. (22, 23). Although there were no significant differences between groups in the incidences of clinical events (including non-fatal reinfarction, target vessel revascularization, and cardiac death) during the 30-day follow-up period, it should be noted that our study would have had insufficient power to detect differences between groups because the rates of these clinical events were low.

Recombinant human pro-UK was selected for use in the present study because it is a fibrin-specific fibrinolytic drug associated with a higher patency rate and fewer bleeding complications than streptokinase (25, 26). The dose of pro-UK (20 mg) used for intracoronary infusion in our study was around 40% of the usual total dose given for intravenous fibrinolytic therapy of myocardial infarction (a bolus dose of 20 mg administered over a 3-min period followed by the infusion of 30 mg during the next 30 min). The dose of streptokinase (250 kU) administered by intracoronary infusion in the studies of Sezer et al. was also lower than the total dose usually given intravenously in patients with myocardial infarction because of the high bleeding risk associated with this agent (22, 23). Another randomized clinical trial utilized low-dose alteplase (10–20 mg) for intracoronary administration (21). The dose of pro-UK used in the present study was selected based on the results of previous investigations, which suggested that local administration of 10–20 mg pro-UK could promote thrombus dissolution and improve myocardial perfusion without increasing the incidence of major bleeding events (38, 39).

In order to reduce the risk of bleeding complications in this study, patients with risk factors for bleeding were excluded, and the PCI procedures were performed via the radial artery. The low rates of TIMI major and minor bleeding events in the pro-UK group (3.6%) and tirofiban group (2.6%) were within the expected range for primary PCI, although most of the patients received antiplatelet therapy (ticagrelor) and an anticoagulant (70–100 U/kg unfractionated heparin) (21, 40). This potent antithrombotic regimen was chosen to alleviate the prothrombotic effects of fibrinolytic therapy, which have been reported for facilitated PCI using tenecteplase or reteplase (41, 42).

A high thrombus burden is an independent predictor of MACEs and infarct-related arterial stent thrombosis in patients treated with stents for STEMI (43). Although routine thrombus aspiration is not recommended during primary PCI, thrombus aspiration may be considered in cases where a large residual thrombus burden is encountered after opening the vessel with the wire or balloon (9, 12). About 80% of the patients in our trial had high-grade thrombus (TIMI 4–5) in the culprit artery, and around half the patients underwent manual thrombus aspiration, which is a higher proportion than that reported in other studies of intracoronary tirofiban or fibrinolytic therapy (21–23, 38, 39). Importantly, thrombus aspiration was not associated with stroke or transient ischemic attack in our trial. We speculate that the relatively high rate of thrombus aspiration in our study may have contributed to the high rate of final TIMI grade 3 flow despite the high thrombus burden.

The timing and positioning of intracoronary fibrinolytic therapy remains controversial. In the present clinical trial, targeted intracoronary infusion of the study drug proximal to the culprit lesion was intended to limit the fibrinolytic effect to the high-grade luminal thrombus, reduce any systemic actions of the drug and minimize bleeding events. However, previous studies have used differing approaches. Sezer et al. infused 250 kU streptokinase through the guiding catheter for 3-min immediately after post-procedural coronary angiography (22). Geng et al. administered 10 mg pro-UK into the distal end of the culprit lesion via a balloon catheter after balloon dilatation (38). McCartney et al. selectively infused alteplase into the infarct-related artery proximal to the culprit lesion over a 5–10-min period before stent implantation (21). Further research is needed to determine whether intracoronary fibrinolytic therapy should be performed before or after arterial patency has been achieved and proximal or distal to the culprit lesion.

This study has some limitations. First, the follow-up period was only 30 days, so data for longer-term outcomes are not available. Second, echocardiography and ECG aren't sensitive enough for the evaluation of microvascular obstruction and infarct size, which makes it difficult to evaluate the possible mechanisms. In our study, we chose ST-segment resolution as a straightforward and sensitive measure of tissue perfusion. Nevertheless, more accurate assessments of microvascular obstruction and infarct size (e.g., using late gadolinium-enhanced magnetic resonance imaging) may be needed in future investigations. Third, our study had insufficient power to detect differences in efficacy between the pro-UK and tirofiban groups because of the sample size limitation. Finally, the dose and timing of intracoronary fibrinolytic therapy need to be clarified, since these factors were not evaluated in our trial.

## Conclusions

Adjunctive intracoronary administration of pro-UK or tirofiban before stent implantation during primary PCI may improve myocardial reperfusion without increasing the incidence of major bleeding events.

## Data Availability Statement

The raw data supporting the conclusions of this article will be made available by the authors, without undue reservation.

## Ethics Statement

The studies involving human participants were reviewed and approved by Ethics committee of Zhongshan Hospital affiliated to Fudan University (No. B2012-134). The patients/participants provided their written informed consent to participate in this study.

## Author Contributions

DH: methodology, writing, and data analysis. JQ: methodology, supervision, and review & editing. ZL: investigation and data analysis. YX, XZ, ZQ, WF, LJ, WH, CS, CL, and QZ: investigation. JG: conceptualization, methodology, and supervision. All authors contributed to the article and approved the submitted version.

## Conflict of Interest

The authors declare that the research was conducted in the absence of any commercial or financial relationships that could be construed as a potential conflict of interest.

## Publisher's Note

All claims expressed in this article are solely those of the authors and do not necessarily represent those of their affiliated organizations, or those of the publisher, the editors and the reviewers. Any product that may be evaluated in this article, or claim that may be made by its manufacturer, is not guaranteed or endorsed by the publisher.
